# A study on the association between different delivery modes and pelvic floor muscle strength in women during early postpartum period

**DOI:** 10.3389/fgwh.2026.1835125

**Published:** 2026-09-09

**Authors:** Huanyu Lin, Xuhong Lu, Shu Yu, Min Yu

**Affiliations:** 1 Zhangzhou Municipal Hospital of Fujian Province and Zhangzhou Affiliated Hospital of Fujian Medical University, Zhangzhou, China; 2 Affiliated Zhongshan Hospital of Dalian University, Dalian, China

**Keywords:** cesarean section, electrical stimulation, pelvic floor biofeedback, pelvic floor muscle strength, urinary incontinence, vaginal delivery

## Abstract

**Background/objectives:**

Pelvic floor dysfunction (PFD) arises from factors such as degeneration and trauma, leading to weakened pelvic floor fascia, ligaments, perineum, adjacent muscles, and other supporting structures in women. Clinically, PFD manifests as stress urinary incontinence (SUI), pelvic organ prolapse (POP), and abnormal pelvic floor muscle strength, substantially impairing women's health. This study investigated the association between pelvic floor electromyography results in postpartum women at 6–8 weeks after delivery and various pregnancy- and childbirth-related factors, with the aim of identifying determinants of impaired pelvic floor function during the early postpartum period.

**Methods:**

A retrospective analysis was performed on 1,154 postpartum women who delivered at the Obstetrics Department of Zhongshan Hospital, Dalian University, and underwent pelvic floor electromyography 6–8 weeks postpartum. Participants were classified into the vaginal delivery group (*n* = 802) and the cesarean section group (*n* = 352) based on delivery mode. The analysis examined differences in early postpartum pelvic floor muscle strength between the two delivery modes and identified factors influencing pelvic floor muscle strength in each group.

**Results:**

Electrical activity of pelvic floor muscles in both the fast- and slow-twitch phases was lower in the vaginal delivery group compared with the cesarean section group, whereas resting-phase values before and after contraction were higher in the cesarean section group. In the vaginal delivery group, independent risk factors for early postpartum pelvic floor muscle strength impairment included pregnancy weight gain > 16 kg, second-stage labor duration ≥ 2 h, advanced maternal age at first birth, episiotomy, perineal laceration, and gestational hypertension. In the cesarean section group, pregnancy weight gain ≥ 11.5 kg, pre-pregnancy BMI > 24 kg/m², gestational hypertension, premature rupture of membranes, preeclampsia, history of uterine fibroids, and full-term delivery were identified as independent risk factors.

**Conclusions:**

Both delivery modes are associated with changes in early-postpartum pelvic-floor muscle electromyographic activity. Altered EMG suggestive of impaired muscle activation is observed more prominently among women after vaginal delivery, while greater changes in resting pelvic-floor muscle tone are noted in the cesarean-delivery group. Cesarean delivery, therefore, does not eliminate the risk of impaired pelvic-floor muscle function or the development of PFD.

## 1 Introduction

The female pelvic floor constitutes an integrated anatomical unit comprising multiple layers of muscles and fascia enclosing the pelvic outlet, through which the urethra, vagina, and rectum traverse. Under normal physiological conditions, pelvic floor components—including muscles, fascia, and ligaments—provide structural support to the uterus, bladder, rectum, and other pelvic organs, thereby maintaining their anatomical alignment. Pelvic floor disorders (PFD) arise from factors such as degenerative changes or trauma that weaken these supporting structures in women (1). Clinical manifestations include urinary incontinence (UI), fecal or anal incontinence (FI or AI), vaginal wall prolapse, pelvic organ prolapse (POP), sexual dysfunction (SD), and chronic pelvic pain, with UI and POP being the most prevalent (2). The pathogenesis of PFD involves relaxation or injury of pelvic floor support systems, with pregnancy and childbirth recognized as independent risk factors (3). This association stems from the mechanical and biochemical alterations during pregnancy: sustained traction exerted by the enlarged uterus on pelvic floor structures may cause direct mechanical damage, while pregnancy- and delivery-related hormonal fluctuations modify collagen metabolism within pelvic connective tissues, thereby promoting the initiation and progression of PFD (4, 5).

Pelvic floor muscle strength serves as a key electrophysiological marker for early detection of pelvic floor function, given its strong correlation with the number of activated motor units, where motor unit recruitment reflects the surface electromyographic activity of the muscle (6). Limited evidence exists regarding surface electromyographic measurements of anterior and posterior resting potentials across different delivery modes. In a study by Resende et al., no statistically significant difference was observed in baseline activity between pregnant and non-pregnant women (*P* = 0.58), indicating that pelvic floor muscle surface electromyographic activity during the resting phase remains unaffected by pregnancy (7). This finding allows the present analysis to exclude pregnancy-related confounding factors and more accurately assess the influence of delivery mode on anterior and posterior resting phase surface electromyographic values, thereby reflecting pelvic floor muscle tension.

PFD is a multifactorial condition characterized by numerous contributing elements, varied and often inconspicuous symptoms, and a marked reduction in quality of life, resulting in substantial psychological and physical distress for affected women. Advances in medical technology have progressively refined the approaches and techniques for PFD research and diagnosis. Nonetheless, investigations focusing on specific influencing factors remain insufficient and warrant further study. In 1995, Glazer analyzed pelvic floor surface electromyography data from PFD patients, proposed an evaluation protocol, and established an international database for pelvic floor surface electromyography (8). The pelvic floor musculature consists of slow-twitch (Type I) and fast-twitch (Type II) muscle fibers. The Glazer assessment measures the functional performance of both fiber types through sequences of contraction and relaxation, recording electrical potentials during activity and rest to detect abnormalities in muscle activity patterns and accurately identify fiber-specific damage. Additionally, this method supports individualized rehabilitation strategies, which has led to its broad clinical adoption as a valuable PFD assessment tool. In this study, 1,154 postpartum women who delivered in our obstetric department underwent Glazer assessment at 6–8 weeks postpartum. The aim was to determine differences in early pelvic floor muscle strength between delivery modes and to analyze factors influencing early pelvic floor muscle strength in vaginal and cesarean deliveries, thereby providing a robust reference for the treatment and prevention of PFD. Definitions of pelvic floor-related outcomes in the present study follow the ICS-IUGA consensus terminology for obstetric pelvic floor disorders (9).

## 2 Materials and methods

### 2.1 Case collection

From September 2022 to September 2024, a total of 1,154 women who delivered at the Obstetrics Department of Zhongshan Hospital, Dalian University, and underwent pelvic floor muscle strength screening at the pelvic floor clinic 6–8 weeks postpartum, in accordance with the established inclusion and exclusion criteria, were enrolled. Based on delivery mode, 802 were assigned to the vaginal delivery group and 352 to the cesarean section group. Data were extracted from electronic obstetric medical records and pelvic-floor clinic records by two trained researchers with a standardized form. Extraction disagreements were resolved through discussion. Participants with incomplete records were excluded, and no data imputation was conducted. Notably, all participants in this study were women who voluntarily attended postpartum pelvic-floor screening. This sampling feature may lead to selection bias.

### 2.2 Inclusion and exclusion criteria

Inclusion criteria: (1) Singleton pregnancy; (2) Vaginal or cesarean delivery; (3) Voluntary participation in pelvic floor function screening at 6–8 weeks postpartum; (4) Complete clinical records. Exclusion criteria: (1) Multiple pregnancies; (2) Conversion from vaginal to cesarean delivery; (3) History of chronic constipation or persistent cough; (4) Severe cardiac, hepatic, renal, or other systemic diseases; (5) Incomplete clinical records.

### 2.3 Pelvic floor muscle electrical assessment standards

Surface electromyography reflects pelvic-floor muscle electrical activation rather than directly quantifying actual muscle force or muscle-fiber injury. Pelvic floor electromyography was measured via the biofeedback electrical stimulation system (Model SA9800) manufactured by Nanjing Vishee Medical Technology Co., Ltd., and the Glazer assessment protocol was implemented accordingly, comprising (15):

1. Anterior and posterior resting phase values, reflecting pelvic floor muscle tension at rest (normal value < 4 μV; values ≥ 4 μV indicate elevated resting muscle tension);
2. Fast muscle phase values, assessing type II muscle fiber function (maximum value > 40 μV; a test maximum below the lower reference threshold indicates reduced type II fiber strength);
3. Slow muscle phase values, assessing type I muscle fiber function (average value > 35 μV; a test average below the lower reference threshold indicates diminished type I fiber endurance).

#### 2.3.1 Determination method

After the subject emptied their bladder, they were placed in the lithotomy position of the bladder. The specialist doctor put on sterile gloves and inserted a disposable probe of the pelvic floor surface electromyography analysis system into the vagina of the subject. First, the doctor taught the subject to learn to voluntarily contract the target muscles, then instructed the subject to contract and release the vagina with maximum force and speed for 5 times, with the total time within 30 s; then instructed the subject to continuously contract the vagina for 5 times, each lasting for 10 s, and the total process time was 2 min and 35 s.

In the present study, these threshold values were adopted to dichotomize continuous EMG indicators into normal and abnormal outcomes. The categorized binary variables were used as dependent variables in the subsequent logistic regression analysis. All pelvic floor electromyography assessments were completed by clinically trained operators. Blinding to delivery mode was not feasible for this retrospective study.

### 2.4 Observation indicators

**Figure 1:**
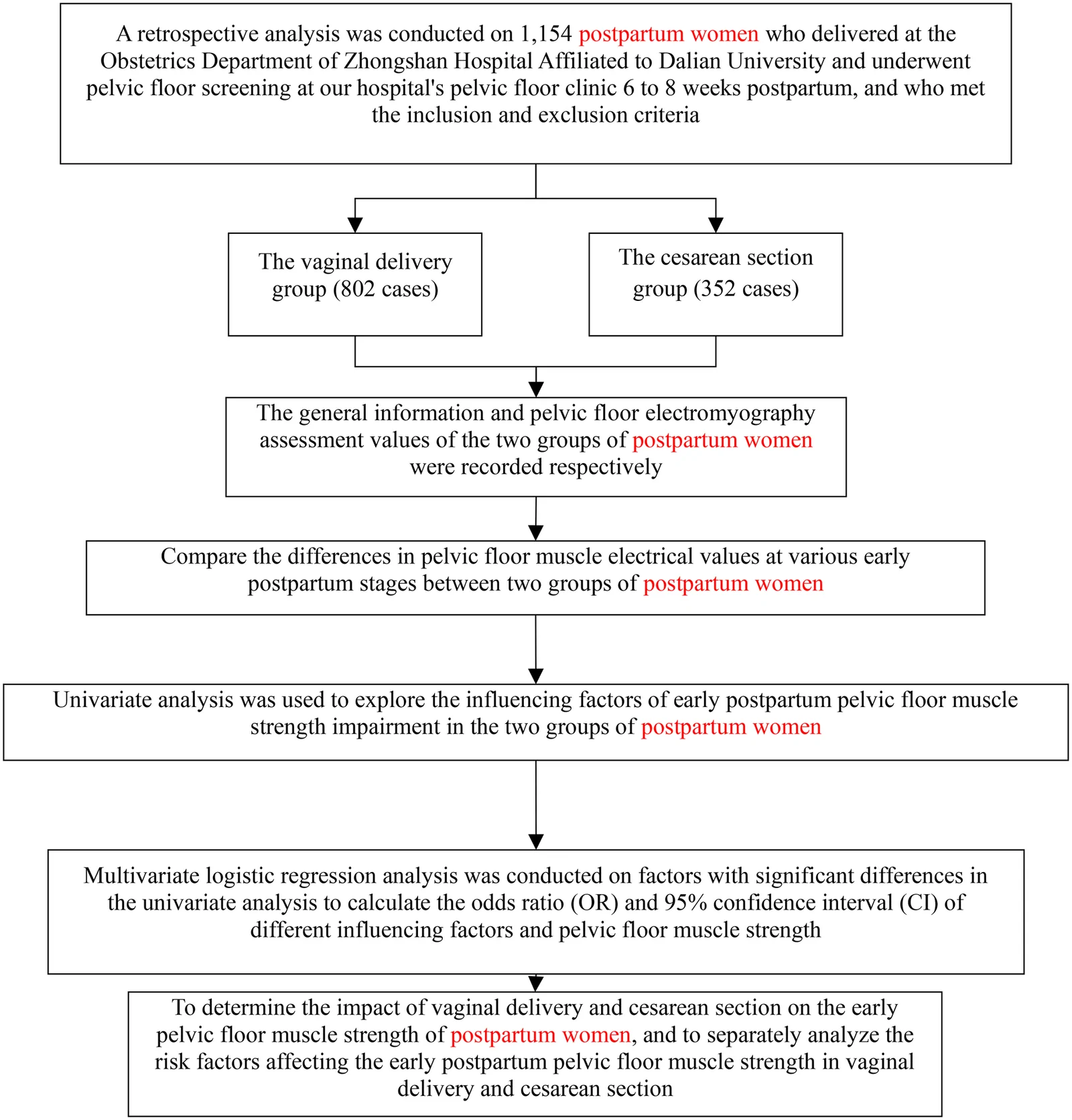
Flow chart.

Participants were categorized into vaginal delivery and cesarean section groups according to delivery mode. Pelvic floor electromyographic values served as the primary evaluation metric to examine the effects of delivery mode on pelvic floor muscle strength. The distribution of potential influencing factors—such as maternal age at delivery, gestational weight gain, neonatal birth weight, and perineal condition at delivery—was analyzed for each stage in both groups. Continuous variables were converted into categorical variables for multivariate logistic regression analysis according to the 2009 Institute of Medicine (IOM) gestational weight gain guidelines, with pre-pregnancy BMI stratified at 24 kg/m^2^ and gestational weight gain stratified at 11.5 kg and 16 kg as the cut-off points (10). Women with a first delivery at age ≥ 35 years were defined as advanced-age nulliparous women (advanced maternal age primiparae). Factors exhibiting statistically significant intergroup differences underwent further multivariate analysis. The technical pathway is illustrated in Figure 1.

### 2.5 Statistical methods

The statistical analyses were performed using SPSS 26.0. Continuous variables were presented as mean ± standard deviation (x̅ ± s) and compared between two groups using the independent-samples *t*-test. Categorical variables were expressed as counts and percentages [*n* (%)], with intergroup comparisons conducted via the chi-square test. Logistic regression analysis was applied to determine factors associated with abnormalities. Statistical significance was defined as *P* *<* 0.05. Prior to the independent-samples *t*-test, the normality of continuous variables was assessed using the Shapiro–Wilk test and histograms, and variance homogeneity was evaluated. Although surface EMG data are frequently skewed in clinical practice, most EMG variables in the present study approximated a normal distribution; hence independent-samples *t*-tests were used. Non-parametric tests would have been adopted for strongly skewed data. This retrospective observational study was reported according to the STROBE guidelines for observational epidemiological research (11).

## 3 Results

### 3.1 The influence of vaginal delivery and cesarean section on early pelvic floor muscle strength in postpartum women

#### 3.1.1 Under normal circumstances

The vaginal delivery group (802 cases) had a mean age of (31.14 ± 3.79) years, BMI of (22.27 ± 3.50) kg/m², gestational weight gain of (15.22 ± 5.29) kg, and neonatal birth weight of (3,434.94 ± 419.16) g. The cesarean section group (352 cases) had a mean age of (32.40 ± 4.03) years, BMI of (23.02 ± 4.06) kg/m², gestational weight gain of (16.53 ± 7.56) kg, and neonatal birth weight of (3,397.33 ± 488.09) g. Statistical analysis revealed no significant differences in maternal age, BMI, gestational weight gain, or neonatal birth weight between the two groups (*P* > 0.05) (Table 1).

**Table 1:** Comparison of general data between the two group.

Variable	Vaginal delivery group (*n* = 802)	Cesarean section group (*n* = 352)	*T*	*P*
Age (years)	31.14 ± 3.79	32.40 ± 4.03	−1.637	0.102
BMI (kg/m^2^)	22.27 ± 3.50	23.02 ± 4.06	−1.856	0.064
Weight gain during pregnancy (kg)	15.22 ± 5.29	16.53 ± 7.56	−1.555	0.121
Newborn weight (g)	3,434.94 ± 419.16	3,397.33 ± 488.09	1.256	0.219

#### 3.1.2 Comparison of electromyographic values at each stage between the vaginal delivery group and the cesarean section group

According to Table 2, inter-group comparisons demonstrated statistically significant differences in electromyography (EMG) values across all examined stages (*P* *<* 0.001). In the vaginal delivery group, EMG values in both the fast muscle and slow muscle stages were lower than those in the cesarean section group, whereas the pre-rest and post-rest stage EMG values in the cesarean section group exceeded those observed in the vaginal delivery group.

**Table 2:** Comparison of EMG values at different stages between vaginal delivery group and cesarean delivery group.

Variable	Vaginal delivery group(*n* = 802)	Cesarean section group(*n* = 352)	*T*	*P*
Electromyography in pre-resting phase	4.62 ± 3.45	7.53 ± 4.45	−10.922	<0.001
Electromyography in post-resting phase	4.21 ± 3.16	7.35 ± 4.43	−12.032	<0.001
Electromyography in fast-twitch muscle phase	34.16 ± 16.89	45.44 ± 18.23	−10.192	<0.001
Electromyography in slow-twitch muscle phase	23.69 ± 14.24	31.28 ± 12.88	−8.578	<0.001

### 3.2 Analysis of factors affecting early pelvic floor muscle strength in women after vaginal delivery and cesarean section

#### 3.2.1 Analysis of factors affecting pelvic floor muscle strength in vaginal delivery

##### 3.2.1.1 Pre-resting phase EMG values

Univariate analysis identified BMI, advanced maternal age at first birth, gestational hypertension, episiotomy, and perineal laceration as potential determinants of pre-resting stage EMG (*P* *<* 0.05), as presented in Table 3. Using the mean EMG value during the pre-resting stage as the dependent variable, and BMI, advanced maternal age at first birth, gestational hypertension, episiotomy, and perineal laceration as independent variables, a multivariate logistic regression was performed. The analysis revealed that gestational hypertension, advanced maternal age at first birth, episiotomy, and perineal laceration independently contributed to abnormal pre-resting EMG values, as summarized in Table 4. The extremely wide 95% CI for advanced-age nulliparous women (OR = 10.146, 95%CI = 1.044–98.639) indicates highly unstable effect estimation. This subgroup only contained 9 participants (1 normal outcome, 8 abnormal outcomes). The result is of limited reference value and should be interpreted with extreme caution.

**Table 3:** Univariate analysis of pelvic floor electromyography in pre-resting phase in vaginal delivery group.

Factors	The normal electromyography value group (*n* = 382)	Abnormal electromyography value group (*n* = 420)	t/*x*^2^	*P*
Age (years)	31.20 ± 3.75	31.08 ± 3.82	0.48	0.633
BMI (kg/m^2^)	21.94 ± 3.36	22.58 ± 3.61	−2.59	0.010
Weight gain during pregnancy (kg)	15.47 ± 5.21	14.99 ± 5.37	1.30	0.194
Second stage of labor (h)	1.02 ± 0.89	0.94 ± 0.76	1.43	0.154
Newborn weight (g)	3,436.52 ± 426.49	3,433.50 ± 412.88	0.10	0.919
Advanced-age nulliparous women *n*(%)	1 (0.26)	8 (1.90)	4.87	0.027
Preeclampsia *n*(%)	12 (3.14)	5 (1.19)	3.67	0.055
Membranes *n*(%)	118 (30.89)	116 (27.62)	1.04	0.309
Diabetes mellitus *n*(%)	68 (17.80)	94 (22.38)	2.60	0.107
Hypertension *n*(%)	16 (4.19)	42 (10.00)	10.07	0.002
Uterine fibroids *n*(%)	22 (5.76)	38 (9.05)	3.13	0.077
Group B strep in pregnancy *n*(%)	28 (7.33)	36 (8.57)	0.42	0.517
Labor epidural analgesia *n*(%)	312 (81.68)	338 (80.48)	0.19	0.665
Threatened preterm labor *n*(%)	18 (4.71)	16 (3.81)	0.40	0.526
Primiparous women *n*(%)	216 (56.54)	252 (60.00)	0.98	0.321
Full-term delivery (≥37 weeks) *n*(%)	374 (97.91)	408 (97.14)	0.48	0.489
Dystocia *n*(%)	126 (32.98)	114 (27.14)	3.26	0.071
Episiotomy *n*(%)	82 (21.47)	245 (58.33)	112.60	<0.001
Perineal laceration *n*(%)	172 (45.03)	300 (71.43)	57.59	<0.001

**Table 4:** Multivariate analysis of pelvic electromyography in pre-resting phase in vaginal delivery group.

Factors	B	SE	Wald	*P*	OR (95%CI)
BMI > 24 kg/m^2^	0.067	0.187	0.129	0.720	1.069 (0.741–1.543)
Hypertension	0.968	0.347	7.788	0.005	2.633 (1.334–5.197)
Advanced-age nulliparous women	2.317	1.160	3.987	0.046	10.146 (1.044–98.639)
Episiotomy	1.925	0.181	113.236	<0.001	6.855 (4.809–9.772)
Perineal laceration	1.508	0.178	72.160	<0.001	4.518 (3.190–6.399)

##### 3.2.1.2 Post-resting phase EMG values

Univariate analysis identified perineal episiotomy and perineal laceration (*P* *<* 0.001) as potential factors associated with post-resting phase EMG values (Table 5). Using the mean post-resting EMG value as the dependent variable and perineal episiotomy along with perineal laceration as independent variables, multivariate logistic regression analysis demonstrated that both conditions independently increased the likelihood of abnormal EMG values in the post-resting phase (Table 6).

**Table 5:** Univariate analysis of pelvic floor electromyography in post-resting phase in vaginal delivery group.

Factors	The normal electromyography value group (*n* = 446)	Abnormal electromyography value group (*n* = 356)	t/*x*^2^	*P*
Age (years)	31.14 ± 3.72	31.13 ± 3.88	0.05	0.958
BMI (kg/m^2^)	22.09 ± 3.44	22.50 ± 3.58	−1.61	0.107
Weight gain during pregnancy (kg)	15.34 ± 5.35	15.06 ± 5.22	0.73	0.464
Second stage of labor (h)	0.97 ± 0.84	0.99 ± 0.80	−0.31	0.755
Newborn weight (g)	3,452.02 ± 409.70	3,413.54 ± 430.33	1.29	0.197
advanced-age nulliparous women *n*(%)	5 (1.12)	4 (1.12)	0.00	>0.99
Preeclampsia *n*(%)	8 (1.79)	9 (2.53)	0.51	0.473
Membranes *n*(%)	132 (29.60)	102 (28.65)	0.09	0.770
Diabetes mellitus *n*(%)	86 (19.28)	76 (21.35)	0.52	0.469
Hypertension *n*(%)	35 (7.85)	23 (6.46)	0.57	0.451
Uterine fibroids *n*(%)	36 (8.07)	24 (6.74)	0.51	0.477
Group B strep in pregnancy *n*(%)	36 (8.07)	28 (7.87)	0.01	0.915
Labor epidural analgesia *n*(%)	358 (80.27)	292 (82.02)	0.40	0.529
Threatened preterm labor *n*(%)	20 (4.48)	14 (3.93)	0.15	0.700
Primiparous women *n*(%)	256 (57.40)	212 (59.55)	0.38	0.539
Full-term delivery (≥37 Weeks) *n*(%)	438 (98.21)	344 (96.63)	2.02	0.155
Dystocia *n*(%)	140 (31.39)	100 (28.09)	1.03	0.311
Episiotomy *n*(%)	100 (22.42)	227 (63.76)	140.12	<0.001
Perineal laceration *n*(%)	206 (46.19)	266 (74.72)	66.55	<0.001

**Table 6:** Multivariate analysis of pelvic electromyography in post-resting phase in vaginal delivery group.

Factors	B	SE	Wald	*P*	OR (95%CI)
Episiotomy	2.221	0.188	140.262	<0.001	9.215 (6.381–13.309)
Perineal laceration	1.756	0.192	83.799	<0.001	5.788 (3.974–8.429)

##### 3.2.1.3 EMG values in the fast muscle phase

Univariate analysis identified the duration of the second stage of labor, episiotomy, and perineal laceration as potential determinants of EMG values (*P* *<* 0.05, Table 7). Using the maximum EMG value in this phase as the dependent variable, with the aforementioned parameters as independent variables, multivariate logistic regression analysis was performed. The results indicated that a second stage of labor lasting ≥ 2 h, episiotomy, and perineal laceration each represented independent risk factors for abnormal EMG values in the fast muscle phase (Table 8).

**Table 7:** Univariate analysis of pelvic floor electromyography in fast-twitch muscle phase in vaginal delivery group.

Factors	The normal electromyography value group (*n* = 260)	Abnormal electromyography value group (*n* = 542)	t/*x*^2^	*P*
Age (years)	30.87 ± 3.96	31.27 ± 3.70	−1.39	0.165
BMI (kg/m^2^)	22.29 ± 3.44	22.26 ± 3.54	0.10	0.918
Weight gain during pregnancy (kg)	14.74 ± 4.94	15.44 ± 5.44	−1.82	0.070
Second stage of labor (h)	0.75 ± 0.69	1.09 ± 0.86	−6.03	<0.001
Newborn weight (g)	3,435.88 ± 418.74	3,434.48 ± 419.75	0.04	0.965
Advanced-age nulliparous women *n*(%)	2 (0.77)	7 (1.29)	0.09	0.765
Preeclampsia *n*(%)	2 (0.77)	15 (2.77)	3.38	0.066
Membranes *n*(%)	78 (30.00)	156 (28.78)	0.13	0.723
Diabetes mellitus *n*(%)	54 (20.77)	108 (19.93)	0.08	0.781
Hypertension *n*(%)	19 (7.31)	39 (7.20)	0.00	0.954
Uterine fibroids *n*(%)	18 (6.92)	42 (7.75)	0.17	0.677
Group B strep in pregnancy *n*(%)	20 (7.69)	44 (8.12)	0.04	0.835
Labor epidural analgesia *n*(%)	204 (78.46)	446 (82.29)	1.67	0.196
Threatened preterm labor *n*(%)	10 (3.85)	24 (4.43)	0.15	0.702
Primiparous women *n*(%)	150 (57.69)	318 (58.67)	0.07	0.792
Full-term delivery (≥37 Weeks) *n*(%)	254 (97.69)	528 (97.42)	0.05	0.815
Dystocia *n*(%)	72 (27.69)	168 (31.00)	0.91	0.339
Episiotomy *n*(%)	66 (25.38)	261 (48.15)	37.73	<0.001
Perineal laceration *n*(%)	116 (44.62)	356 (65.68)	32.20	<0.001

**Table 8:** Multivariate analysis of pelvic electromyography in rapid stage in vaginal delivery group.

Factors	B	SE	Wald	*P*	OR (95%CI)
Second stage of labor ≥ 2 h	1.011	0.301	11.280	0.001	2.747 (1.523–4.955)
Episiotomy	1.054	0.174	36.563	<0.001	2.870 (2.039–4.040)
Perineal laceration	1.046	0.164	40.798	<0.001	2.845 (2.064–3.921)

##### 3.2.1.4 EMG values in the slow muscle phase

Univariate analysis identified weight gain during pregnancy, duration of the second stage of labor, neonatal weight, parity, episiotomy, and perineal laceration as potential factors affecting EMG values (*P* *<* 0.05) (Table 9). Using the maximum EMG value in this phase as the dependent variable, and the above parameters as independent variables, multivariate logistic regression analysis revealed that pregnancy weight gain exceeding 16 kg, a second stage of labor lasting ≥ 2 h, episiotomy, and perineal laceration independently contributed to an increased likelihood of abnormal slow muscle phase (Table 10).

**Table 9:** Univariate analysis of pelvic electromyography in slow-twitch muscle phase in vaginal delivery group.

Factors	The normal electromyography value group (*n* = 116)	Abnormal electromyography value group (*n* = 686)	t/*x*^2^	*P*
Age (years)	30.90 ± 4.21	31.18 ± 3.71	−0.74	0.460
BMI (kg/m^2^)	22.86 ± 3.61	22.17 ± 3.48	1.94	0.053
Weight gain during pregnancy (kg)	13.88 ± 4.70	15.44 ± 5.36	−2.95	0.003
Second stage of labor (h)	0.76 ± 0.67	1.02 ± 0.84	−3.70	<0.001
Newborn weight (g)	3,506.55 ± 406.08	3,422.83 ± 420.41	1.99	0.047
Advanced-age nulliparous women *n*(%)	0 (0.00)	9 (1.31)	0.58	0.445
Preeclampsia *n*(%)	2 (1.72)	15 (2.19)	0.00	>0.99
Membranes *n*(%)	28 (24.14)	206 (30.03)	1.67	0.197
Diabetes mellitus *n*(%)	28 (24.14)	134 (19.53)	1.30	0.253
Hypertension *n*(%)	6 (5.17)	52 (7.58)	0.86	0.354
Uterine fibroids *n*(%)	52 (7.58)	50 (7.29)	0.25	0.614
Group B strep in pregnancy *n*(%)	12 (10.34)	52 (7.58)	1.03	0.310
Labor epidural analgesia *n*(%)	90 (77.59)	560 (81.63)	1.06	0.304
Threatened preterm labor *n*(%)	2 (1.72)	32 (4.66)	1.45	0.228
Primiparous women *n*(%)	58 (50.00)	410 (59.77)	3.89	0.048
Full-term delivery (≥37 Weeks) *n*(%)	114 (98.28)	668 (97.38)	0.06	0.800
Dystocia *n*(%)	30 (25.86)	210 (30.61)	1.07	0.301
Episiotomy *n*(%)	30 (25.86)	297 (43.29)	12.49	<0.001
Perineal laceration *n*(%)	56 (48.28)	416 (60.64)	6.27	0.012

**Table 10:** Multivariate analysis of pelvic electromyography in slow-twitch muscle phase in vaginal delivery group.

Factors	B	SE	Wald	*P*	OR (95%CI)
Weight gain during pregnancy (kg)					
<11.5 kg				1	
11.5–16 kg	0.199	0.256	0.608	0.436	1.220 (0.740–2.014)
>16 kg	0.682	0.270	6.397	0.011	1.978 (1.166–3.355)
Second stage of labor ≥ 2 h	1.131	0.451	6.288	0.012	3.100 (1.280–7.507)
Newborn weight (g)					
≤2,500 g				1	
2,500–3,000 g	1.305	0.927	1.985	0.159	3.689 (0.600–22.677)
3,000–3,500 g	0.089	0.835	0.011	0.915	1.093 (0.213–5.612)
>3,500 g	−0.034	0.839	0.002	0.967	0.966 (0.186–5.007)
Primiparous women *n*(%)	0.118	0.213	0.309	0.578	1.126 (0.742–1.709)
Episiotomy	0.831	0.238	12.173	<0.001	2.295 (1.439–3.660)
Perineal laceration	0.686	0.213	10.384	0.001	1.985 (1.308–3.013)

#### 3.2.2 Analysis of factors affecting pelvic floor muscle strength during cesarean section

##### 3.2.2.1 Pre-resting phase EMG values

Univariate analysis identified BMI, gestational weight gain, gestational hypertension, and preterm birth as variables significantly associated with pre-resting phase EMG measurements (*P* *<* 0.05) (Table 11). Using the mean EMG value in the pre-resting phase as the dependent variable, with BMI, gestational weight gain, gestational hypertension, and preterm birth as independent variables, a multivariate logistic regression analysis was performed. The analysis revealed that BMI > 24 kg/m², gestational weight gain ≥ 11.5 kg, gestational hypertension, and full-term birth were independent risk factors (Table 12).

**Table 11:** Univariate analysis of pelvic floor electromyography in pre-resting phase in cesarean section.

Factors	The normal electromyography value group (*n* = 82)	Abnormal electromyography value group (*n* = 270)	t/*x*^2^	*P*
Age (years)	32.80 ± 3.76	32.28 ± 4.11	1.03	0.304
BMI (kg/m^2^)	21.05 ± 3.42	23.63 ± 4.05	−5.23	<0.001
Weight gain during pregnancy (kg)	12.81 ± 8.11	17.66 ± 7.02	−4.89	<0.001
Newborn weight (g)	3,391.95 ± 548.29	3,398.96 ± 469.39	−0.11	0.909
Advanced-age nulliparous women *n*(%)	10 (12.20)	34 (12.59)	0.01	0.924
Preeclampsia *n*(%)	11 (13.41)	26 (9.63)	0.96	0.328
Membranes *n*(%)	12 (14.63)	28 (10.37)	1.14	0.287
Diabetes mellitus *n*(%)	28 (34.15)	76 (28.15)	1.09	0.297
Hypertension *n*(%)	16 (19.51)	99 (36.67)	8.41	0.004
Scarred uterus *n*(%)	2 (2.44)	20 (7.41)	2.65	0.104
Uterine Fibroids *n*(%)	16 (19.51)	46 (17.04)	0.27	0.606
Group B strep in pregnancy *n*(%)	6 (7.32)	12 (4.44)	0.56	0.454
Labor epidural analgesia *n*(%)	2 (2.44)	10 (3.70)	0.04	0.837
Threatened preterm labor *n*(%)	2 (2.44)	12 (4.44)	0.24	0.623
Primiparous women *n*(%)	44 (53.66)	126 (46.67)	1.23	0.267
Full-term delivery (≥37 Weeks) *n*(%)	72 (87.80)	264 (97.78)	12.21	<0.001

**Table 12:** Multivariate analysis of pelvic floor electromyography in pre-resting phase in cesarean section.

Factors	B	SE	Wald	*P*	OR (95%CI)
BMI > 24 kg/m^2^	1.029	0.314	10.710	0.001	2.798 (1.511–5.182)
Weight gain during pregnancy					
<11.5 kg	1				
11.5–16 kg	1.746	0.382	20.892	<0.001	5.734 (2.712–12.124)
>16 kg	1.676	0.332	25.557	<0.001	5.344 (2.790–10.233)
Hypertension	1.214	0.348	12.143	<0.001	3.368 (1.701–6.668)
Full-term delivery	1.538	0.606	6.444	0.011	4.657 (1.420–15.275)

##### 3.2.2.2 Post-resting phase EMG values

Univariate analysis identified BMI, gestational weight gain, premature rupture of membranes, and full-term delivery as variables significantly associated with post-resting phase EMG values (*P* *<* 0.05) (Table 13). Using the mean post-resting phase EMG value as the dependent variable, with BMI, gestational weight gain, premature rupture of membranes, and full-term delivery as independent variables, multivariate logistic regression revealed that gestational weight gain ≥ 11.5 kg, premature rupture of membranes, and full-term delivery functioned as independent risk factors for abnormal post-resting phase EMG (Table 14).

**Table 13:** Univariate analysis of pelvic floor electromyography in post-resting phase in cesarean section group.

Factors	The normal electromyography value group (*n* = 88)	Abnormal electromyography value group (*n* = 264)	t/*x*^2^	*P*
Age (years)	32.70 ± 4.36	32.30 ± 3.92	0.81	0.420
BMI (kg/m^2^)	20.81 ± 4.12	23.76 ± 3.77	−6.21	<0.001
Weight gain during pregnancy (kg)	13.81 ± 8.14	17.43 ± 7.15	−3.98	<0.001
Newborn weight (g)	3,328.41 ± 528.23	3,420.30 ± 472.79	−1.53	0.126
Advanced-age nulliparous women *n*(%)	10 (11.36)	34 (12.88)	0.14	0.710
Preeclampsia *n*(%)	8 (9.09)	29 (10.98)	0.25	0.616
Membranes *n*(%)	4 (4.55)	36 (13.64)	5.42	0.020
Diabetes mellitus *n*(%)	32 (36.36)	72 (27.27)	2.62	0.106
Hypertension *n*(%)	24 (27.27)	91 (34.47)	1.55	0.213
Scarred uterus *n*(%)	6 (6.82)	16 (6.06)	0.06	0.799
Uterine fibroids *n*(%)	10 (11.36)	52 (19.70)	3.16	0.076
Group B strep in pregnancy *n*(%)	6 (6.82)	12 (4.55)	0.31	0.576
Labor epidural analgesia *n*(%)	2 (2.27)	10 (3.79)	0.12	0.734
Threatened preterm labor *n*(%)	4 (4.55)	10 (3.79)	0.00	>0.99
Primiparous women *n*(%)	36 (40.91)	134 (50.76)	2.56	0.109
Full-term delivery (≥37 weeks) *n*(%)	80 (90.91)	256 (96.97)	4.28	0.039

**Table 14:** Multivariate analysis of pelvic floor electromyography in post-resting phase in cesarean section.

Factors	B	SE	Wald	*P*	OR (95%CI)
BMI > 24 kg/m^2^	0.271	0.278	0.955	0.328	1.312 (0.761–2.261)
Weight gain during pregnancy					
<11.5 kg	1				
11.5–16 kg	0.881	0.330	7.133	0.008	2.413 (1.264–4.605)
>16 kg	1.384	0.313	19.574	<0.001	3.990 (2.161–7.365)
Membranes	1.532	0.620	6.096	0.014	4.627 (1.371–15.613)
Full-term delivery	1.318	0.606	4.726	0.030	3.736 (1.139–12.260)

##### 3.2.2.3 EMG values in the fast muscle phase

Univariate analysis identified BMI and preeclampsia as potential determinants of EMG values in the fast muscle phase (*P* *<* 0.05, Table 15). Using the mean EMG value of the fast muscle phase as the dependent variable, and BMI together with preeclampsia as independent variables, multivariate logistic regression revealed that BMI > 24 kg/m² and preeclampsia were independent risk factors for abnormal fast muscle phase EMG (Table 16).

**Table 15:** Univariate analysis of pelvic floor electromyography in rapid muscle phase in cesarean section group.

Factors	The normal electromyography value group (*n* = 214)	Abnormal electromyography value group (*n* = 138)	t/*x*^2^	*P*
Age (years)	32.11 ± 4.04	32.86 ± 4.00	−1.69	0.092
BMI (kg/m^2^)	22.60 ± 4.21	23.69 ± 3.73	−2.47	0.014
Weight gain during pregnancy (kg)	16.34 ± 7.82	16.82 ± 7.15	−0.58	0.563
Newborn weight (g)	3,407.66 ± 504.68	3,381.30 ± 462.54	0.49	0.622
Advanced-age nulliparous women *n*(%)	24 (11.21)	20 (14.49)	0.82	0.364
Preeclampsia *n*(%)	16 (7.48)	21 (15.22)	5.34	0.021
Membranes *n*(%)	20 (9.35)	20 (14.49)	2.21	0.137
Diabetes mellitus *n*(%)	64 (29.91)	40 (28.99)	0.03	0.853
Hypertension *n*(%)	76 (35.51)	39 (28.26)	2.01	0.157
Scarred uterus *n*(%)	14 (6.54)	8 (5.80)	0.08	0.778
Uterine fibroids *n*(%)	36 (16.82)	26 (18.84)	0.24	0.627
Group B strep in pregnancy *n*(%)	12 (5.61)	6 (4.35)	0.27	0.600
Labor epidural analgesia *n*(%)	8 (3.74)	4 (2.90)	0.02	0.902
Threatened preterm labor *n*(%)	10 (4.67)	4 (2.90)	0.69	0.406
Primiparous women *n*(%)	102 (47.66)	68 (49.28)	0.09	0.768
Full-term delivery (≥37 weeks) *n*(%)	206 (96.26)	130 (94.20)	0.82	0.365

**Table 16:** Multivariate analysis of pelvic electromyography in rapid stage in cesarean section.

Factors	B	SE	Wald	*P*	OR (95%CI)
BMI > 24 kg/m^2^	0.570	0.223	6.525	0.011	1.769 (1.142–2.740)
Preeclampsia n(%)	0.808	0.355	5.171	0.023	2.243 (1.118–4.501)

##### 3.2.2.4 EMG values in the slow muscle phase

Univariate analysis indicated a significant association between uterine fibroids and EMG values (*P* *<* 0.05, Table 17). Subsequent multivariate logistic regression, with the mean EMG value of the slow muscle phase as the dependent variable and uterine fibroids as the independent variable, confirmed uterine fibroids as an independent risk factor for slow muscle phase abnormalities (Table 18).

**Table 17:** Univariate analysis of pelvic floor electromyography in slow muscle phase in cesarean section group.

Factors	The normal electromyography value group (*n* = 120)	Abnormal electromyography value group (*n* = 232)	t/*x*^2^	*P*
Age (years)	31.83 ± 3.72	32.70 ± 4.17	−1.91	0.056
BMI (kg/m^2^)	22.79 ± 4.70	23.15 ± 3.69	−0.72	0.474
Weight gain during pregnancy (kg)	15.97 ± 8.74	16.82 ± 6.87	−0.92	0.360
Newborn weight (g)	3,371.83 ± 494.09	3,410.52 ± 485.51	−0.70	0.482
Advanced-age nulliparous women *n*(%)	12 (10.00)	32 (13.79)	1.04	0.308
Preeclampsia *n*(%)	8 (6.67)	29 (12.50)	2.86	0.091
Membranes *n*(%)	14 (11.67)	26 (11.21)	0.02	0.897
Diabetes mellitus *n*(%)	38 (31.67)	66 (28.45)	0.39	0.530
Hypertension *n*(%)	46 (38.33)	69 (29.74)	2.65	0.103
Scarred uterus *n*(%)	4 (3.33)	18 (7.76)	2.64	0.104
Uterine fibroids *n*(%)	14 (11.67)	48 (20.69)	4.44	0.035
Group B strep in pregnancy *n*(%)	6 (5.00)	12 (5.17)	0.00	0.945
Labor epidural analgesia *n*(%)	4 (3.33)	8 (3.45)	0.00	>0.990
Threatened preterm labor *n*(%)	4 (3.33)	10 (4.31)	0.02	0.875
Primiparous women *n*(%)	50 (41.67)	120 (51.72)	3.20	0.073
Full-term delivery (≥37 weeks) *n*(%)	116 (96.67)	220 (94.83)	0.62	0.432

**Table 18:** Multivariate analysis of pelvic electromyography in slow-twitch muscle phase in cesarean section group.

Factors	B	SE	Wald	*P*	OR (95%CI)
Uterine Fibroids *n*(%)	0.681	0.327	4.324	0.038	1.975 (1.040–3.752)

## 4 Discussion

It should be noted that surface electromyography captures muscle electrical activity, which is correlated with muscle activation, but cannot directly measure muscle force, tissue integrity, or actual fiber-level damage. In recent years, the incidence of PFD has shown a sustained upward trend, with its high prevalence and low medical consultation rate emerging as a significant global public health concern. PFD includes a spectrum of complex disorders, including UI, dysuria, POP, FI, dyspareunia, and chronic pelvic pain (12), affecting women at various stages of life, substantially impairing quality of life, and often necessitating prolonged management. Understanding the etiological factors and pathophysiological mechanisms of early postpartum PFD is essential for effective prevention and intervention. The etiology in women is multifactorial, involving variables such as age, pregnancy, BMI, prior pelvic surgery, mode of vaginal delivery, and pre-existing pelvic conditions (13). Early impairment of pelvic floor function may present as structural abnormalities in pelvic floor muscle fibers, progressing to overt clinical manifestations of PFD. The pelvic floor comprises two principal fiber types: type I and type II muscle fibers. Type I fibers are slow-twitch, capable of isotonic contraction, and constitute the deep pelvic musculature. They exhibit resistance to fatigue, sustain prolonged contraction, and maintain resting pelvic floor tone to ensure structural stability and organ closure; damage to these fibers predisposes to POP. Type II fibers are fast-twitch, capable of isometric contraction, and fatigue more readily. They generate rapid contractions in response to increased intra-abdominal pressure to preserve organ closure and swiftly retract excretory channels during elimination, serving key roles in the control of urination, defecation, and sexual function (14, 15).

Pelvic floor surface electromyography (sEMG) quantifies the electrical activity and reflex responses of pelvic floor muscles. Vaginal electrode measurements capture electromyographic signals from both superficial and deep muscle layers, where potential values correspond to the number of activated motor units and are proportional to contraction strength, thereby indirectly indicating muscle power (16). Glazer's assessment framework currently serves as the most widely applied quantitative standard for pelvic floor sEMG, providing a structured protocol that objectively evaluates the function of type I (slow-twitch) and type II (fast-twitch) muscle fibers (17). In this study, pelvic floor muscle sEMG was evaluated according to the Glazer protocol. The Glazer sEMG protocol is widely applied for evaluating postpartum pelvic-floor muscle function. Previous published studies have supported its acceptable reliability for assessing pelvic-floor muscle activity among women at 6–8 weeks postpartum (17). However, intra-rater and inter-rater reliability analyses were not conducted in the present single-center cohort, which should be considered when interpreting our results. EMG values recorded during pre-rest and post-rest stages reflect baseline muscle tone, with elevated values in these stages indicating increased resting tension. Measurements obtained during the fast muscle phase assess type II fiber function, whereas values from the slow muscle phase indicate type I fiber performance. Importantly, the sEMG parameters in the present study reflect pelvic-floor muscle bioelectrical activity rather than clinical symptomatic pelvic floor dysfunction. As validated symptom questionnaires and standardized pelvic floor physical examinations were not available for this retrospective cohort, observed inter-group differences in electromyography should not be directly extrapolated to clinical outcomes such as urinary incontinence, pelvic organ prolapse, fecal incontinence, sexual dysfunction, pelvic pain, or quality-of-life impairment. It should be noted that inconsistent findings exist across previous studies regarding obstetric risk factors for postpartum pelvic-floor muscle electrophysiology. As this is an observational retrospective study, significant associations reported herein cannot establish causality. The mechanistic interpretations presented below represent hypothetical explanations that require further validation in future investigations.

The study results suggest that delivery mode is associated with differences in early postpartum pelvic floor function. Relative to cesarean section, greater injury to both type I and type II muscle fibers is observed among women with vaginal delivery, rendering them more susceptible to damage. In contrast, higher pre- and post-resting EMG values were observed in the cesarean delivery group. These elevated resting electrical activities may be attributable to multiple potential factors, such as insufficient muscle relaxation, postoperative discomfort, psychological anxiety, or measurement variability, and should not be simply interpreted as definitive clinical pelvic floor dysfunction. Both delivery modes are associated with PFD-related manifestations. However, owing to the lack of data on cesarean-section indications, indication bias cannot be excluded when interpreting these inter-group differences. Previous studies have identified cesarean section as a risk factor for PFD when compared with Primiparous women (18). MacArthur et al. (19) reported that up to 40% of women who delivered by cesarean section developed UI within 12 years postpartum. Furthermore, certain PFDs exhibit a prolonged latency, with Thomas et al. (20) observing an average interval of 33.5 years from levator ani impaired muscle function to POP surgery in primiparas. Given these delayed manifestations, the long-term impact of delivery mode on pelvic floor function warrants further investigation through multicenter, large-sample prospective cohort studies. Moreover, the EMG cut-offs adopted in this study originate from general female reference data; dedicated validation for early-postpartum women with our device is lacking, which should be kept in mind when interpreting our categorical results.

This study showed that episiotomy and perineal laceration were identified as independent risk factors for early postpartum pelvic floor muscle strength impairment in the vaginal delivery group. Episiotomy involves an incision of the perineal tissue before fetal head crowning to expedite delivery and reduce the risk of laceration, and it remains one of the most frequently performed surgical interventions in women (21). Evidence regarding its protective effect on pelvic floor function remains inconclusive. A systematic review screening 986 articles and including 26 relevant studies evaluating episiotomy outcomes reported no protective benefit of episiotomy against PFD manifestations such as FI, POP, SUI, or reduced pelvic floor muscle strength (22). Additionally, postoperative complications including perineal pain, edema, induration, and direct injury to pelvic floor muscles and ligaments may adversely affect sexual quality of life (23). Given the interplay of multiple delivery-related factors, most available evidence is derived from observational studies, and randomized controlled trials remain challenging. The influence of episiotomy on pelvic floor function thus warrants further investigation.

This analysis demonstrated that a second stage of labor lasting ≥ 2 h constituted an independent risk factor for early postpartum pelvic floor muscle strength impairment in women undergoing vaginal delivery. The second stage spans from complete cervical dilation to fetal expulsion, during which uterine contractions and maternal pushing drive the fetal head downward, imposing substantial mechanical load on pelvic floor structures and inducing varying degrees of distension, stretching, and deformation of the soft tissues. It is hypothesized that prolongation or arrest of this stage is correlated with increased mechanical stress, which may be linked to irreversible injury of the pelvic floor musculature and postpartum PFD (24). Vaginal delivery necessitates enlargement of the levator ani hiatus to permit fetal passage; however, this expansion induces levator ani muscle relaxation, predisposing to muscle or nerve injury and elevating the risk of postpartum POP (25, 26). Greater degrees of levator ani hiatus enlargement correlate with more severe functional impairment and a markedly increased likelihood of POP (27). To facilitate fetal descent through the birth canal, pelvic floor muscles undergo substantial elongation during vaginal delivery, particularly in primiparous women, a mechanism identified by Lien et al. (28). The present results align with these observations, indicating that an extended duration of the second stage is associated with higher likelihood of pelvic floor muscle strength deterioration.

This study found that weight gain exceeding 16 kg during pregnancy emerged as an independent risk factor for abnormal pelvic floor muscle strength in the vaginal delivery group during the early postpartum period, whereas a pre-pregnancy BMI above 24 kg/m² combined with pregnancy weight gain of at least 11.5 kg constituted independent risk factors in the cesarean section group. Elevated pre-pregnancy BMI is associated with a higher incidence of PFD after childbirth, likely due to prolonged mechanical loading on pelvic floor tissues prior to conception. Sustained compression and traction from excess adipose tissue in the waist and abdomen may reduce tissue elasticity, which is associated with increased susceptibility to PFD. With advancing gestational age, excessive pregnancy weight gain further elevates intra-abdominal pressure, which may correlate with structural and functional damage to pelvic floor muscles, ligaments, and nerves. Such changes not only increase the likelihood of cesarean delivery but also heighten the risk of adverse pregnancy outcomes (29). Excess abdominal adipose tissue during delivery can diminish the supportive contribution of abdominal and diaphragmatic muscle contractions to expulsive force, thereby necessitating stronger contractions of both abdominal and pelvic floor muscles and intensifying the strain on pelvic floor structures. Furthermore, excessive abdominal fat exerts persistent traction on pelvic floor muscles and associated nerves, promoting sustained overextension, elevating pelvic organ pressure and mobility, and contributing to postpartum PFD (30). Harrison CL reported that each additional kilogram of gestational weight gain was associated with a 10% increase in adverse pregnancy outcomes (31). Conversely, inadequate weight gain during pregnancy may also predispose to PFD, as insufficient adipose tissue and muscular support reduce cushioning and protective capacity, increasing vulnerability to pelvic floor injury. Given its modifiable nature, maintaining appropriate gestational weight is essential for minimizing pregnancy-related pelvic floor dysfunction.

Hypertensive disorders during pregnancy pose significant threats to maternal and neonatal health, ranking among the leading causes of morbidity and mortality in pregnant women and perinatal infants. The present study identified gestational hypertension as an independent risk factor for abnormal pre-resting stage pelvic floor muscle EMG values in both vaginal and cesarean deliveries. Physiologically, placental development begins with blastocyst implantation into the endometrium, followed by extra villous trophoblast migration through the decidual matrix toward the spiral arteries. This process involves interactions with endothelial and smooth muscle cells of the spiral arteries, generating signaling molecules that mediate immune, inflammatory, endocrine, and metabolic exchanges between mother and placenta, thereby modulating maternal adaptation to pregnancy (32). In gestational hypertension, One possible explanation is that excessive inflammatory activity can induce a cascade of complications, variably affecting pelvic floor structures and tissues. Given the functional integration of pelvic floor vasculature, neural pathways, and musculature, such alterations are likely to be associated with early postpartum pelvic floor muscle strength decline (33), although the precise mechanisms underlying this injury remain to be elucidated. No prior studies have examined the association between preeclampsia and postpartum pelvic floor muscle strength. The present analysis identified preeclampsia as an independent risk factor for abnormal fast muscle phase EMG values in cesarean section patients, potentially linked to hypertension-induced multi-organ ischemia and hypoxia. Preeclampsia represents a chronic hypoxic condition of pregnancy, characterized by elevated blood pressure after 20 weeks of gestation, with a reported incidence of approximately 5%–10% (34). Further investigations are warranted to elucidate its relationship with postpartum pelvic floor muscle strength impairment. Additionally, a history of uterine fibroids emerged as an independent risk factor for abnormal slow muscle phase EMG values in cesarean section patients. Uterine fibroids constitute the most common benign neoplasms of the female reproductive tract, affecting an estimated 25%–40% of women of reproductive age (35). No studies have documented the association between uterine fibroids and PFD; however, A potential hypothesis is that uterine fibroids are hormone-dependent tumors strongly influenced by estrogen and progesterone, with peak incidence between 30 and 50 years, spanning most of the reproductive period. Dysregulated hormone secretion may contribute to the elevated incidence of postpartum PFD. In the present study, premature rupture of premature rupture of membranes was identified as an independent risk factor for abnormal pelvic floor electromyography parameters during the post-resting phase in patients undergoing cesarean section. This condition results from the interplay of multiple factors and may precipitate complications such as preterm birth, umbilical cord prolapse, and puerperal infection (36). Evidence regarding its association with PFD remains limited, potentially due to the irregular uterine contractions and abnormal labor patterns it induces, which can aggravate vaginal lacerations in postpartum women. Similarly, no research has established a link between full-term delivery and PFD, indicating the need for further investigation to clarify these relationships.

## 5 Conclusion

1. Postpartum injury to both type I and type II pelvic floor muscle fibers is more significant following vaginal delivery than after cesarean section, whereas resting pelvic floor muscle tone is higher in the cesarean section group. Consequently, cesarean section does not entirely prevent postpartum PFD and should be undertaken solely when clinically indicated.
2. In the vaginal delivery group, independent risk factors for early postpartum pelvic floor muscle strength impairment include gestational weight gain > 16 kg, a second stage of labor ≥ 2 h, advanced maternal age at first birth, episiotomy, perineal laceration, and gestational hypertension. In the cesarean section group, such risk factors include gestational weight gain ≥ 11.5 kg, pre-pregnancy BMI > 24 kg/m², gestational hypertension, premature rupture of premature rupture of membranes, preeclampsia, history of uterine fibroids, and full-term delivery. Pregnant women with these high-risk profiles should receive intensified prenatal monitoring and management.

### 5.1 Limitation

This study has Five main limitations. First, we did not report model fit statistics, discriminative performance metrics, multicollinearity diagnosis results, or multiplicity correction for all logistic regression models. The pre-specified statistical analysis plan only included standard logistic regression without supplementary model evaluation and multiple testing adjustment procedures, which may slightly raise the risk of type I error during variable screening. Second, the subgroup of advanced-age nulliparous women contained merely nine participants, leading to an unstable odds ratio with an extremely wide confidence interval. Though Firth's penalized logistic regression could mitigate sparse-data bias, we could not implement this extra statistical approach under the original analytical framework. We have added clear cautionary notes in the Results section and Table 4 to remind readers that this association result is exploratory rather than definitive. Future large-sample research is required to validate the relevant correlation. Third, the comparisons between vaginal delivery and cesarean delivery were unadjusted. Maternal age, BMI, pregnancy complications and surgical indications for cesarean section may act as potential confounding factors. Further multivariate adjusted analyses are required in future prospective studies to validate our findings. Fourth, this was a single-center retrospective study. Participants were restricted to women who attended pelvic floor function screening at 6–8 weeks after delivery. Therefore, the results cannot be generalized to all postpartum women, and multi-center research is needed to verify our conclusions in larger and more diverse populations. In addition, information regarding clinical indications for cesarean section was not fully available in our retrospective electronic medical records; hence, we could not adjust for indication bias. Since cesarean delivery is non-randomized and closely related to maternal-fetal conditions, the inter-group comparison of pelvic-floor electromyography results between vaginal and cesarean groups should be interpreted cautiously. Fifth, selection bias may exist because only women who voluntarily attended postpartum pelvic-floor screening were enrolled. Women receiving screening may differ from non-attenders in pelvic-floor symptoms, health-seeking motivation, socioeconomic status and obstetric risk. Therefore, our results may not fully represent the whole postpartum population.

## Data Availability

The original contributions presented in the study are included in the article/Supplementary Material, further inquiries can be directed to the corresponding author/s.
