# Supplementary material for: A study on the association between different delivery modes and pelvic floor muscle strength in women during early postpartum period
Source: Front Glob Womens Health. 2026 Sep 9;7:1835125. doi: 10.3389/fgwh.2026.1835125 (PMC13597690; doi:10.3389/fgwh.2026.1835125)
Supplement: Supplementary file 1 [file Datasheet1.pdf]

STROBE Statement—checklist of items that should be included in reports of observational studies

	Item No	Recommendation
**Title and abstract**	**1**	**(a) Indicate the study's design with a commonly used term in the title or the abstract**<br>Filled: Page 1 Abstract / Title<br>Notes: Abstract clearly states "A retrospective analysis was performed on 1,154 parturients", study design: retrospective cross-sectional observational study<br>**(b) Provide in the abstract an informative and balanced summary of what was done and what was found**<br>Filled: Page 1 Abstract<br>Notes: Abstract covers background, methods, grouping, main EMG results, independent risk factors, conclusions
**Introduction**		
**Background/rationale**	**2**	**2 Background/rationale: Explain the scientific background and rationale for the investigation being reported**<br>Filled: Page 2–3 Introduction<br>Notes: Introduce PFD hazards, Glazer pelvic floor EMG evaluation, research gaps of delivery mode on early postpartum pelvic floor muscle function
**Objectives**	**3**	**3 Objectives: State specific objectives, including any prespecified hypotheses**<br>Filled: Page 3 last paragraph of Introduction<br>Notes: Clear objective: Compare pelvic floor EMG differences between vaginal and cesarean delivery; analyze independent risk factors for pelvic floor dysfunction in two groups
**Methods**		
**Study design**	**4**	**4 Study design: Present key elements of study design early in the paper**<br>Filled: Page 4 Section 2.1 Case collection & Page7 Section2.5 Statistical methods<br>Notes: Retrospective cross-sectional observational study, single-center obstetric cohort, 6–8 weeks postpartum Glazer assessment
**Setting**	**5**	**5 Setting: Describe the setting, locations, and relevant dates, including periods of recruitment, exposure, follow-up, and data collection**<br>Filled: Page4 Section2.1 Case collection<br>Notes: Setting: Obstetrics Department of Zhongshan Hospital, Dalian University; recruitment period: September 2022 to September 2024; follow-up: 6–8 weeks postpartum pelvic floor screening
**Participants**	**6**	**6 Participants**<br>**Cross-sectional study —Give the eligibility criteria, and the sources**

		**and methods of selection of participants**<br>Filled: Page4 Section2.1 & 2.2 Inclusion and exclusion criteria<br>Notes: Clear inclusion criteria (singleton, vaginal/cesarean delivery, complete records, 6–8w screening); exclusion criteria (multiple pregnancy, conversion delivery, chronic cough/constipation, severe systemic disease, incomplete data)
**Variables**	**7**	**7 Variables: Clearly define all outcomes, exposures, predictors, potential confounders, and effect modifiers. Give diagnostic criteria, if applicable**<br>Filled: Page4–5 Section2.3, 2.4 Observation indicators<br>Notes:<br>Primary outcome: Glazer EMG values (pre-rest, post-rest, fast muscle, slow muscle phase) with normal threshold criteria;<br>Exposure variable: delivery mode (vaginal / cesarean);<br>Confounders/predictors: maternal age, BMI, gestational weight gain, neonatal birth weight, episiotomy, perineal laceration, gestational hypertension, preeclampsia, premature rupture of membranes, uterine fibroids etc.
**Data sources/ measurement**	**8***	**8 Data sources/ measurement: For each variable of interest, give sources of data and details of methods of assessment (measurement). Describe comparability of assessment methods if there is more than one group**<br>Filled: Page4 Section2.3 Pelvic floor muscle electrical assessment standards<br>Notes: All participants adopted unified Glazer surface electromyography detection protocol; consistent detection standards for vaginal and cesarean groups, unified normal reference thresholds for all EMG phases
**Bias**	**9**	**9 Bias: Describe any efforts to address potential sources of bias**<br>Filled: Page4 Section2.2 exclusion criteria & Page7 statistical methods<br>Notes: Excluded patients with chronic pelvic interference factors; multivariate logistic regression used to adjust confounding factors to reduce selection bias and confounding bias
**Study size**	**10**	**10 Study size: Explain how the study size was arrived at**<br>Filled: Page4 Section2.1 Case collection<br>Notes: All eligible primiparous women receiving routine pelvic floor screening from Sep 2022–Sep 2024 were consecutively enrolled, total n=1154 without pre-specified sample size calculation (retrospective consecutive sampling)
**Quantitative variables**	**11**	**11 Quantitative variables: Explain how quantitative variables were handled in the analyses. If applicable, describe which groupings were chosen and why**<br>Filled: Page7 Section2.5 Statistical methods & all univariate/multivariate tables<br>Notes: Continuous variables (age, BMI, gestational weight gain, EMG

		values) expressed as mean ± SD; categorical variables grouped by clinical cut-off values: BMI > 24, gestational weight gain ≥11.5kg / > 16kg, second stage labor ≥2h, full-term delivery ≥37 weeks, divided into subgroups for regression analysis
**Statistical methods**	**12**	**12 Statistical methods**<br>**(a) Describe all statistical methods, including those used to control for confounding**<br>**(b) Describe any methods used to examine subgroups and interactions**<br>**(c) Explain how missing data were addressed**<br>**(d) Cross-sectional study —If applicable, describe analytical methods taking account of sampling strategy**<br>**(e) Describe any sensitivity analyses**<br>Filled: Page7 Section2.5 Statistical methods<br>Notes:<br>(a) Independent t-test for continuous variables; chi-square test for categorical variables; multivariate logistic regression to adjust confounders;<br>(b) Subgroup analysis stratified by vaginal delivery / cesarean delivery;<br>(c) Only patients with complete clinical and EMG data were enrolled; cases with missing data excluded during recruitment;<br>(d) Consecutive retrospective sampling of hospital delivery population;<br>(e) No sensitivity analysis performed
**Results**		
**Participants**	**13***	**13 Participants**<br>**(a) Report numbers of individuals at each stage of study—eg numbers potentially eligible, examined for eligibility, confirmed eligible, included in the study, completing follow-up, and analysed**<br>**(b) Give reasons for non-participation at each stage**<br>**(c) Consider use of a flow diagram**<br>Filled: Page4 Section2.1 & Page5 Figure1 Flow chart<br>Notes: Total screened primiparous women n=1154 fully eligible; 802 vaginal delivery, 352 cesarean delivery; Figure 1 provided as participant flow diagram; patients with incomplete records or meeting exclusion criteria were excluded before final enrollment
**Descriptive data**	**14***	**14 Descriptive data**<br>**(a) Give characteristics of study participants (eg demographic, clinical, social) and information on exposures and potential confounders**<br>**(b) Indicate number of participants with missing data for each variable of interest**<br>**(c) Cohort study —Summarise follow-up time (eg, average and total amount)**<br>Filled: Page8 Table1 general data comparison<br>Notes: Demographic data (age, BMI, gestational weight gain, newborn weight) of two groups listed in Table1; no missing data in enrolled

		participants; follow-up uniformly at 6–8 weeks postpartum for all subjects
**Outcome data**	**15***	**15 Outcome data**<br>**Cross-sectional study— Report numbers of outcome events or summary measures**<br>Filled: Page9 Table2 EMG value comparison between two groups<br>Notes: Mean ± SD of pre-rest, post-rest, fast muscle, slow muscle EMG values reported for vaginal and cesarean groups; normal/abnormal EMG subgroup counts listed in all univariate analysis tables (Table3, Table15 etc.)
**Main results**	**16**	**16 Main results**<br>**(a) Give unadjusted estimates and, if applicable, confounder-adjusted estimates and their precision (eg, 95% confidence interval). Make clear which confounders were adjusted for and why they were included**<br>**(b) Report category boundaries when continuous variables were categorized**<br>**(c) If relevant, consider translating estimates of relative risk into absolute risk for a meaningful time period**<br>Filled: All univariate tables (Table3, Table11 etc.) & multivariate logistic regression tables (Table4, Table12, Table16 etc.)<br>Notes: Univariate crude P values; multivariate adjusted OR and 95%CI; confounders selected based on clinical relevance and univariate P<0.05; clear cut-off boundaries for stratified continuous variables (BMI 24, gestational weight gain 11.5kg/16kg, second stage labor 2h); absolute risk not calculated
**Other analyses**<br><br>**Discussion**	**17**	**17 Other analyses: Report other analyses done—eg analyses of subgroups and interactions, and sensitivity analyses**<br>Filled: Page9–17 Section3.2 Subgroup analysis by delivery mode<br>Notes: Separate univariate and multivariate regression for vaginal delivery subgroup and cesarean delivery subgroup; no interaction or sensitivity analysis
**Key results**	**18**	**18 Key results: Summarise key results with reference to study objectives**<br>Filled: Page18–23 Section4 Discussion opening paragraph<br>Notes: Recap core EMG differences between two delivery modes and all independent risk factors, match preset research objectives
**Limitations**	**19**	**19 Limitations: Discuss limitations of the study, taking into account sources of potential bias or imprecision. Discuss both direction and magnitude of any potential bias**<br>Filled: Page23 Discussion middle paragraph<br>Notes: Single-center retrospective cross-sectional design; only short-term 6–8w postpartum indicators observed, lacking long-term follow-up data; cannot confirm causal relationship, only correlation; limited

		generalizability to other hospitals/regions
**Interpretation**	**20**	**20 Interpretation: Give a cautious overall interpretation of results considering objectives, limitations, multiplicity of analyses, results from similar studies, and other relevant evidence**<br>Filled: Full Section4 Discussion<br>Notes: Compare results with existing published literature; reasonably interpret risk factor mechanisms; cautiously explain research limitations, avoid over-interpreting causal inference
**Generalisability**	**21**	**21 Generalisability: Discuss the generalisability (external validity) of the study results**<br>Filled: Page23 Discussion limitation part<br>Notes: Subjects are single-center primiparous women; results may not fully apply to multipara, different ethnic populations or hospitals with different screening protocols
**Other information**		
**Funding**	**22**	**22 Funding: Give the source of funding and the role of the funders for the present study and, if applicable, for the original study on which the present article is based**<br>Filled: N/A<br>Notes: No funding information provided in the manuscript

*Give information separately for cases and controls in case-control studies and, if applicable, for exposed and unexposed groups in cohort and cross-sectional studies.

Note: An Explanation and Elaboration article discusses each checklist item and gives methodological back ground and published examples of transparent reporting. The STROBE checklist is best used in conjuncti on with this article (freely available on the Web sites of PLoS Medicine at http://www.plosmedicine.org/, Annals of Internal Medicine at http://www.annals.org/, and Epidemiology at http://www.epidem.com/). Infor mation on the STROBE Initiative is available at www.strobe-statement.org.
