# Supplementary material for: A study on the association between different delivery modes and pelvic floor muscle strength in women during early postpartum period
Source: Front Glob Womens Health. 2026 Sep 9;7:1835125. doi: 10.3389/fgwh.2026.1835125 (PMC13597690; doi:10.3389/fgwh.2026.1835125)
Supplement: Supplementary file 2 [file Table1.docx]

**Title Page**

**Title：**A Study on the Impact of Different Delivery Methods on Early Postpartum Pelvic Floor Muscle Strength in Women

**Authors:** Huanyu Lin^1^，Min Yu^2^

**Affiliations:**

^1^Zhangzhou Municipal Hospital of Fujian Province,Zhangzhou, Fujian, China

^2^Affiliated Zhongshan Hospital of Dalian University, Dalian,Liaoning, China

***: Corresponding Author:**

Yu Min，MD，Affiliations : Affiliated Zhongshan Hospital of Dalian University, Dalian,Liaoning, China.E-mail: **[Yucorn2000@126.com](mailto:Yucorn2000@126.com)**

**Conflict of interest statement：**The authors declare no competing interests.
**Authors and affiliations**

**1. Huanyu Lin, MD**

**Affiliations:** Zhangzhou Municipal Hospital of Fujian Province,Zhangzhou, Fujian, China. E-mail:18650202371@163.com

**2. Min Yu, MD**

**Affiliations:** Affiliated Zhongshan Hospital of Dalian University, Dalian,Liaoning, China. E-mail: [Yucorn2000@126.com](mailto:Yucorn2000@126.com)

**Declarations**

**Data availability**

The datasets generated during the current study are not publicly available due to the consent process but are available from the corresponding author on reasonable request.

**Conflict of interest**

The authors declare no competing interests.

**Ethics approval and consent to participate**

All steps of this study were in accordance with the Declaration of Helsinki, and our study was approved by the Research Ethics Committee of Zhongshan Hospital, Dalian University (KY2023-208-1). The researchers were able to maintain and respect the researchers maintained and respected the privacy, confidentiality and well-being of the participants by Not writing down the names or other identifying information of the participants. All data will be used only for the purposes of this study. Written informed consent was obtained from all participants. Written informed consent was obtained from all participants.

**Funding**

No funding.
