# Supplementary material for: A study on the association between different delivery modes and pelvic floor muscle strength in women during early postpartum period
Source: Front Glob Womens Health. 2026 Sep 9;7:1835125. doi: 10.3389/fgwh.2026.1835125 (PMC13597690; doi:10.3389/fgwh.2026.1835125)
Supplement: Supplementary file 3 [file Table2.docx]

**Cover Letter**

Dear Editors of Human Reproduction,

We are writing to submit our original research article entitled “A Study on the Impact of Different Delivery Methods on Early Postpartum Pelvic Floor Muscle Strength in Women” for your consideration for publication in Frontiers in Global Women's Health. This manuscript has not been published previously, nor is it under review for publication in any other journal. All authors have approved the final version of the manuscript and declare no conflicts of interest related to this research.

Pelvic floor dysfunction (PFD) is a highly prevalent complication in postpartum women, severely impairing maternal physical and mental health and quality of life. Pregnancy and childbirth are well-recognized key risk factors for PFD, but the differential impacts of vaginal delivery and cesarean section on pelvic floor muscle (PFM) injury, as well as their specific risk factors, remain not fully elucidated in clinical research. Notably, there is a common clinical misconception that cesarean section can completely avoid PFM injury and PFD occurrence, which lacks sufficient empirical evidence support. To address these clinical gaps, our study conducted a large-sample retrospective analysis to clarify the distinct characteristics of PFM damage caused by different delivery methods and identify their independent risk factors for early postpartum PFM strength impairment, aiming to provide evidence-based clinical guidance for the prevention, screening and intervention of postpartum PFD.

In this study, we enrolled 1,154 primiparous women who delivered at the Affiliated Zhongshan Hospital of Dalian University from September 2022 to September 2024 and underwent pelvic floor electromyography (EMG) screening 6–8 weeks postpartum. According to the delivery mode, the participants were divided into the vaginal delivery group (n=802) and the cesarean section group (n=352). We strictly controlled confounding factors by setting rigorous inclusion and exclusion criteria (e.g., excluding multiple pregnancies, vaginal-to-cesarean conversion, and systemic diseases). Pelvic floor muscle function was evaluated by the internationally recognized Glazer assessment criteria, with EMG indicators of pre-resting phase, post-resting phase, fast-twitch phase and slow-twitch phase as the primary outcome measures. We used independent-samples t-test and chi-square test for univariate analysis, and multivariate Logistic regression analysis to screen the independent risk factors for early postpartum PFM strength impairment in each group, ensuring the scientific rigor and reliability of the research design and statistical methods.

Our key findings revealed significant differences in the characteristics of PFM damage between the two delivery methods: the vaginal delivery group had significantly lower EMG values in both fast and slow-twitch phases, indicating more severe injury to type I and type II PFM fibers; while the cesarean section group had higher EMG values in the resting phases, suggesting abnormal elevation of PFM resting tension. Importantly, our study confirmed that cesarean section does not eliminate the risk of PFM injury and PFD. We further identified the independent risk factors for PFM strength impairment in each group: for vaginal delivery, the risk factors included gestational weight gain >16 kg, second-stage labor duration ≥2 h, advanced maternal age at first birth, episiotomy, perineal laceration, and gestational hypertension; for cesarean section, the risk factors included gestational weight gain ≥11.5 kg, pre-pregnancy BMI >24 kg/m², gestational hypertension, premature rupture of membranes, preeclampsia, history of uterine fibroids, and full-term delivery. Notably, preeclampsia and a history of uterine fibroids were first identified as independent risk factors for PFM strength impairment in cesarean section women, which supplemented the research evidence in this field and provided new insights for clinical stratified management of pregnant women.

This study contributes to the existing literature with three key novel points: first, it clarifies the differentiated damage characteristics of vaginal delivery and cesarean section on early postpartum PFM function, providing a clear empirical basis for correcting the clinical misconception that "cesarean section can prevent PFD"; second, it identifies the specific independent risk factors for PFM strength impairment in different delivery modes through large-sample statistical analysis, especially the newly discovered risk factors in the cesarean section group, which enriches the understanding of the etiology of postpartum PFD; third, the research results provide targeted and evidence-based clinical recommendations for the prevention of postpartum PFD, such as strict control of gestational weight gain, standardized management of hypertensive disorders in pregnancy, rational grasp of the second stage of labor and episiotomy indications, and strict implementation of cesarean section clinical indications. All these findings have important clinical translational value and can directly guide clinical prenatal care, intrapartum management and postpartum pelvic floor rehabilitation work.

Frontiers in Global Women's Health is a prestigious journal in the field of obstetrics and gynecology/urogynecology, with a strong focus on publishing high-quality clinical research that addresses clinical hotspots and has practical application value. Our research is highly consistent with the journal's scope and publishing orientation, and we believe that our findings will provide valuable academic reference for your readers and contribute to the clinical prevention and management of postpartum PFD.

We have attached the full manuscript, all supporting tables and figures, and ethical approval documents for your review. We are willing to revise the manuscript thoroughly and promptly according to the comments and suggestions from the editorial team and reviewers. Thank you for your time and consideration of our submission. We look forward to your favorable response.

Sincerely yours,

Huanyu Lin！
