# Supplementary material for: A study on the association between different delivery modes and pelvic floor muscle strength in women during early postpartum period
Source: Front Glob Womens Health. 2026 Sep 9;7:1835125. doi: 10.3389/fgwh.2026.1835125 (PMC13597690; doi:10.3389/fgwh.2026.1835125)
Supplement: Supplementary file 4 [file Table3.docx]

**Highlights**

1. A large-sample retrospective analysis of 1154 primiparas clarified differentiated pelvic floor muscle (PFM) injury characteristics of vaginal delivery and cesarean section at 6–8 weeks postpartum, with vaginal delivery causing severe Type I/II fiber damage and cesarean section leading to abnormal resting PFM tension.

2. Cesarean section was confirmed to not eliminate the risk of PFM injury and pelvic floor dysfunction (PFD), correcting the clinical misconception that cesarean section can prevent postpartum PFD.

3. Specific independent risk factors for early postpartum PFM strength impairment were identified for each delivery mode, with gestational hypertension as the common risk factor across both groups.

4. Preeclampsia and a history of uterine fibroids were first identified as independent risk factors for PFM impairment in the cesarean section group, supplementing novel research evidence for the field.

5. Targeted clinical recommendations for postpartum PFD prevention were proposed, including stratified management of high-risk pregnant women, strict control of gestational weight gain, and standardized intrapartum intervention, providing evidence-based guidance for clinical practice.
